# Real-Time Dialysis Dose: Ionic Dialysis Versus Classical Urea Kinetic Modeling Indices

**DOI:** 10.7759/cureus.69928

**Published:** 2024-09-22

**Authors:** Diana D Nenova, Yanko G Yankov, Gergana M Chausheva

**Affiliations:** 1 Clinic of Nephrology and Dialysis, University Hospital St. Marina, Varna, BGR; 2 Department of Internal Disease, Medical University "Prof. Dr. Paraskev Stoyanov", Varna, BGR; 3 Clinic of Maxillofacial Surgery, University Hospital St. Marina, Varna, BGR; 4 Department of General and Operative Surgery, Medical University "Prof. Dr. Paraskev Stoyanov", Varna, BGR; 5 Central Clinical Laboratory, University Hospital St. Marina, Varna, BGR; 6 Department of Clinical Laboratory, Medical University "Prof. Dr. Paraskev Stoyanov", Varna, BGR

**Keywords:** adequacy, clearance, conductivity, dialysis dose, diffusion gradient, ionic, online monitoring, sodium, total body water, volume

## Abstract

Introduction

Worldwide, over 850 million people need renal replacement therapy, and over 80% of them are on hemodialysis. The term "dialysis adequacy" is most often associated with the achievement of minimally acceptable indices - single pool Kt/V (spKt/V) and urea reduction ratio (URR%) and largely does not take into account other clinical indicators in patients. In addition, it should be taken into account that in the conditions of clinical practice, their measurement is carried out according to standards controlled by the regulatory structures and is carried out with a frequency between one and three months, and not during each dialysis procedure. Accordingly, based on the obtained value, we assume that it refers to the urea clearance of the entire dialysis prescription. A new methodology for measuring dialysis adequacy through ionic dialysis allows dose estimation in real time without the need for additional blood tests by recording the difference in sodium ion conductivity at the dialyzer inlet and outlet. The latter corresponds to the dialysis dose delivered and can be measured at each dialysis session, enabling timely therapeutic intervention at minimal cost to the community. The aim of this original article is to present ion dialysis to the community as a reliable and inexpensive alternative to classical urea kinetic modeling (UKM), evaluating the comparability of the two methods and the possible sources of error in the result.

Material and methods

This was a retrospective study of 32 patients undergoing hemodialysis at the Clinic of Nephrology and Dialysis at the University Hospital St. Marina (Varna, Bulgaria) for the period between January and December 2020. For each of the patients, four measurements (every three months) of the delivered dialysis dose were performed by ion dialysis and classical urea kinetic modeling in order to assess the comparability and reliability of the two methods.

Results

The analysis of the results proved a high correlation between the validated indicators of dialysis adequacy (spKt/V; URR) and those registered with online monitoring (online clearance monitor) by ionic dialysis - online Kt/V (onKt/V) while at the same time reporting a significant difference in the two methods - primarily based on the anthropometric formulas used to estimate the volume. The established regression models confirmed the high predictive value of ionic dialysis in relation to the actually delivered dose.

Discussion

Despite the high bond strength, our recorded values ​​for onKt/V are 8% lower compared to results using UKM. Therefore, onKt/V as an assessment metric has the ability to underestimate the dialysis dose received. Various factors as a possible source of error and its cause have been reported by some authors. Several studies have reported differences of 2-5% in instantaneous conductance measurements, which they associate mainly with differences in the diffusion coefficients of urea and sodium, as well as different effects of dialysis membrane charge or inadequate ultrafiltration correction.

Conclusions

Our study confirms that online clearance monitoring (OCM) is a practical non-invasive tool for daily use that complements the classic performance of OCM by helping to deliver an adequate dialysis dose with increased patient benefit and minimal cost of financial resources.

## Introduction

Dialysis adequacy and the need for its quantitative measurement have been a subject of discussion since the dawn of hemodialysis treatment. Until the introduction of the principles of urea kinetic modeling (UКM), a clear criterion for evaluating the dialysis dose was lacking. Up to this point, the quality of hemodialysis was evaluated subjectively by the self-esteem of the patients and the absence of uremic pericarditis [1]. However, hemodialysis is a prescribed clinical action, which means that it is essential to use indexes to control dialysis adequacy and accurately assess the planned and obtained result. The term "dialysis adequacy" is most commonly associated with achieving a minimally acceptable index - spKt/V and largely ignores other clinical parameters in patients with end-stage renal disease (ESRD). The recommended minimum dialysis dose, according to the current Kidney Disease Quality Outcome Initiative (KDOQI) standard, is spKt/V ≥1.2 with a target of 1.4 (KDOQI Guidelines 2015) [2]. It should be taken into account that its most frequent measurement is carried out once a month, and, according to the standards in some countries, every three months and based on the value obtained for the relevant dialysis session, it is assumed that the result is representative of the urea clearance of the entire dialysis prescription. Theoretically, this is so, but it should be borne in mind that dialysis adequacy depends on too many factors, such as dialysis time, blood flow rate and dialysate flux, area, and permeability of the dialyzer. While some of these are constant quantities, most require modification in the course of the dialysis procedure according to the individual characteristics of the patient. Therefore, the claim that a single dialysis dose measurement is indicative of all sessions is false. Data from the HEMO trial showed that 21% of patients with a prescribed target spKt/V=1.3 actually had spKt/V<1.2 at some point [3-5].

The ability to monitor dialysis doses in real time is promising for optimal clinical outcomes. The first commercially available technology used the electrical conductivity of the outgoing dialysis solution. These devices take advantage of the fact that sodium fluxes across the dialyzer membrane are an excellent analog of urea clearance and can be measured by changes in the conductance of the dialysate [3,6,7]. This enables an inexpensive and readily available online assessment of dialysis adequacy and sodium clearance that can be measured during each dialysis session. Ionic dialysis is based on measuring the conductivity of the dialysis solution (equivalent to the sodium concentration in the dialysate) at the inlet and recording the change in the outlet conductivity. Estimation of urea clearance by this methodology is considered optimal due to the similar kinetics of urea and sodium, which have comparable molecular weights and volumes of distribution. Transient changes in dialysate conductance recorded during the session are believed not to affect the outcome precisely because of the large volume of sodium distribution [6,8-11]. The method uses the transient increase in dialysate conductivity up to 15.5 mS/cm, corresponding to a sodium concentration in the dialysate of approximately 155 mEq/L. As a result of sodium diffusion into the blood due to this high concentration gradient between the two spaces - dialysate and blood, the dialysate conductivity at the outlet drops. The recorded change in conductance compared to baseline is used to estimate the effective ionic dialysance corresponding to the dialysis dose delivered [6].

The aim of the conducted research is to evaluate the comparability and reliability of the ion dialysis methodology for evaluating the received dialysis dose with the classical indices of urea kinetic modeling (UKM) through the results of online monitoring and blood urea clearance. This would allow the development and implementation in clinical practice of new therapeutic strategies for low-cost assessment of dialysis doses in real time without the need for blood tests.

## Materials and methods

This was a retrospective one-year study conducted during the period from the beginning of January 2020 to the end of December 2020, in which 32 hemodialysis patients at the Clinic of Nephrology and Dialysis, University Hospital St. Marina (Varna, Bulgaria) were studied, compared, and analyzed. It was conducted in accordance with the Declaration of Helsinki.

As part of the study, the medical records of a total of 96 patients were checked, of which 62 met the conditions of the study. Of these, 32 patients who met the inclusion/exclusion criteria were subject to analysis and statistical processing in the present study. A total of 30 patients with an average age of 52±3.8 years (41.2% women and 58.8% men) were excluded from the considered cohort. Seven of them had an adverse event-death during the study; five of them had an implanted pacemaker (interference with the reading of the online clearance monitor); eight had data on hyperhydration due to noncompliance with the prescribed regimen; and in 10 of them, there were problems with vascular access in the form of recirculation or thrombosis, necessitating the placement of a temporary vascular catheter. The latter usually provided a lower flow rate, which would significantly affect the results. The studied cohort did not include patients under the age of 18, as they were dialyzed in specialized pediatric structures.

The inclusion criteria for the study were that the patients had reached 18 years of age, had been on chronic dialysis treatment for a period of more than six months with depleted residual renal function, had signed informed consent to participate in the study, and had permanent vascular access ensuring sufficient blood flow.

Exclusion criteria included patients under 18 years of age, those who had not signed an informed consent to participate in the study, patients with problematic vascular access (with impaired flow rate and/or recirculation), and patients with data on hyperhydration, which could affect ionic conduction.

For each patient, four measurements were performed (every three months) of the classical indicators of dialysis adequacy - spKt/V and URR%, calculated by UKM based on routine blood tests in the clinic, and those of on Kt/V, registered by online monitoring using ionic dialysis for the same sessions. For this purpose, Fresenius Medical Care series 5008 devices (Fresenius Medical Care AG & Co, Germany) were used to determine the received dialysis dose based on ionic dialysis using online clearance monitor (OCM). The technology is standardized but requires pre-setting the value of the urea volume of distribution for the specific patient. This value is calculated according to Watson's formula for total body water (TBW; Table 1). Hemodialysis (HD) was performed according to a conventional schedule three times per week with low-flow polysulfone dialyzers-series "Diadema" (Etropal JSC, Etropole, Bulgaria) with a surface area tailored to the patient's body size. The procedures were carried out for four hours at a blood flow rate (Qb) of 280 ml/min and a dialysate flow (Qd) of 500 ml/min, with a set temperature of 37°C and without sodium profiling in the dialysis solution in order to exclude interference of the results. The data obtained from the online monitoring were meticulously entered into the patient files for each session.

**Table 1:** Mathematical methods t - dialysis time in hours (h); UF - the volume of ultrafiltration in liters (l); W - post-dialysis weight of the patient in kilograms (kg); ln - natural logarithm; Co - pre-dialysis urea nitrogen; C - post-dialysis urea nitrogen; R - C/Co; Age - age in years; Height - height in centimeters (cm); Weight - optimal weight in kilograms (kg); TBW - total body water

Indicator	Formula
spKt/V	spKt/V=​​-ln(R-0.008xt)+(4-3.5xR)x0.55UF/W
Urea reduction ratio% (URR)	URR%=100x(1–C/Co)
TBW men	TBW=2.447-0.09516xAge+0.1074xHeight+0.3362xWeight
TBW women	TBW=-2.097+0.1069xHeight+0.2466xWeight

Laboratory tests for biochemistry (urea, creatinine) in all studied patients were performed routinely according to the requirements of the National Health Insurance Fund of the Republic of Bulgaria. Blood samples were collected under standardized conditions. Urea concentration (mmol/l) was measured by a coupled enzyme reaction with glutamate dehydrogenase (GLDH) using a UV kinetic method on the ADVIA Chemistry 1800 system (Siemens, Germany), with reference ranges of 3.2-8.2 mmol/l. Creatinine concentration (mmol/l) was measured using the Jaffe-kinetic method on the same system, with reference ranges of 44-115 mmol/l. The stop-pump technique was used before and/or after HD to avoid the effect of recirculation and minimize the effect of urea rebound. Blood samples for urea and creatinine were tested both before and at the end of the dialysis procedure to assess dialysis adequacy indicators: spKt/V-index and URR%. The latter were calculated based on the formulas presented in Table 1.

Statistical analysis of the data was performed using SPSS Statistics for Windows, Version 20.0 (IBM Corp., Armonk, New York, United States) on Windows 10 software (Microsoft Corporation, Redmond, Washington, United States). The methods used included descriptive analysis to establish the average levels and variations in quantitative variables, as well as absolute and relative values in qualitative variables. Parametric methods for hypothesis testing (Student's t-test), correlation analysis to study the relationship between the observed phenomena (Pearson's r), and regression analysis with a 95% confidence interval (CI) for the slope of regression were also employed. The results at p<0.05 were considered statistically significant.

## Results

Table 2 presents the individual values from 128 parallel measurements of classical spKt/V (measured by UKM), URR%, and onKt/V (measured based on ionic dialysis) in 32 patients over a period of one year (n=128). In each patient, a total of four measurements were taken at an interval of three months. The mean age of the patients was 51±8.2 years, with 43% women (n=13) and 57% men (n=19).

**Table 2:** Individual values of spKt/V, URR% and onKt/V in the 32 studied patients spKt/V - single pool Kt/V, dialysis adequacy index; URR% - urea reduction ratio, dialysis adequacy index; onKt/V - online Kt/V, dialysis adequacy index, measured by ionic dialysis

	First measurement			Second measurement			Third measurement			Fourth measurement		
No	spKt/V	URR%	onKt/V	spKt/V	URR%	onKt/V	spKt/V	URR%	onKt/V	spKt/V	URR%	onKt/V
1.	1.39	70.3	1.28	1.46	73.8	1.31	1.43	74.6	1.38	1.32	70.6	1.21
2.	1.53	74.1	1.38	1.61	78.7	1.47	1.5	76.4	1.39	1.42	73.5	1.36
3.	1.61	80.2	1.46	1.48	78.6	1.39	1.51	75.3	1.46	1.38	70.4	1.26
4.	1.45	72.1	1.31	1.38	69.5	1.21	1.51	76.7	1.38	1.38	76.5	1.24
5.	1.5	76.4	1.38	1.46	71.3	1.28	1.56	78.2	1.41	1.61	75.4	1.48
6.	1.68	78	1.51	1.5	77.2	1.38	1.38	70.6	1.26	1.23	66.5	1.1
7.	1.23	66.3	1.12	1.2	66.8	1.1	1.32	69.8	1.24	1.41	71.6	1.36
8.	1.06	62	0.9	1.1	59.6	0.9	1	53.4	0.88	1.2	65.2	1
9.	1.23	65.8	1.1	1.3	72.6	1.24	1.42	70.6	1.36	1.44	74.6	1.36
10.	1.1	59.6	0.8	1.2	65.8	1.16	1.35	73.9	1.24	1.46	71.3	1.38
11.	1.35	71.2	1.21	1.41	72.5	1.34	1.36	69.8	1.23	1.28	67.6	1.18
12.	1.51	74.2	1.38	1.45	73.6	1.28	1.42	74.2	1.34	1.44	76.5	1.38
13.	0.8	53.01	0.71	1	58.6	0.9	1.21	67.2	1	1.28	69.4	1.2
14.	1.1	52.6	0.89	1.23	68.3	1.18	1.31	69.3	1.24	1.38	70.6	1.2
15.	1.8	80.4	1.58	1.53	76.5	1.38	1.42	73.6	1.38	1.44	72.3	1.42
16.	1.1	60.2	1	1.24	67.2	1.1	1.28	66.9	1.1	1.26	65.3	1.2
17.	1.65	76.5	1.43	1.51	73.6	1.44	1.41	71.6	1.33	1.38	69.3	1.3
18.	1.7	70.3	1.52	1.63	77.6	1.38	1.4	72.3	1.28	1.38	73.2	1.28
19.	1.2	64.8	1	1.26	67.4	1.2	1.33	74.3	1.24	1.41	73.6	1.37
20.	1.39	68.5	1.28	1.37	74.9	1.3	1.42	75.3	1.3	1.38	72.6	1.32
21.	1.21	63.4	1	1.26	67.2	1.12	1.35	73.6	1.26	1.42	75.2	1.36
22.	1	59.8	0.83	1.18	62.3	1.1	1.25	67.1	1.11	1.3	70.9	1.24
23.	1.75	77.1	1.58	1.64	75.2	1.41	1.52	76.9	1.47	1.43	71.8	1.36
24.	1.2	63.02	0.98	1.26	67.1	1.2	1.36	70.4	1.28	1.29	69.8	1.24
25.	1.15	61.05	0.9	1.1	58.9	0.9	1.27	66.3	1.2	1.31	69.9	1.27
26.	1.35	71.4	1.26	1.41	72.9	1.28	1.38	71.7	1.31	1.32	72.6	1.26
27.	1.22	67.1	1.18	1.24	66.9	1.2	1.3	69.8	1.26	1.38	73.2	1.31
28.	1.52	76.2	1.38	1.44	70.3	1.29	1.36	68.5	1.24	1.42	74.2	1.38
29.	1.4	72.6	1.32	1.38	71.9	1.33	1.36	68.8	1.29	1.42	73.6	1.35
30.	1.35	75	1.21	1.38	71.6	1.26	1.44	73.4	1.32	1.38	72.3	1.31
31.	1.58	78.93	1.42	1.48	77.2	1.32	1.51	78.2	1.4	1.36	69.9	1.21
32.	1.1	60.45	0.89	1.23	66.4	1.18	1.28	66.2	1.2	1.33	70.2	1.27

The results of the variation analysis for the performed 128 measurements of the classical indicators of dialysis adequacy by UKM showed that the obtained average value of spKt/V was 1.36±0.16 with a variation of 0.025 (spKt/V=1.36±0.16, V=0.025, n=128). The mean value of URR was 70.54±5.58% with a variation of 31.15 (URR=70.54±5.58%, V=31.15, n=128). The mean value for parallel measurements by ionic dialysis (onKt/V) was 1.25±0.17 with a variation of 0.028 (onKt/V=1.25±0.17, V=0.028, n=128). The mean value of the Watson-calculated urea volume of distribution (TBW) was 28±4.37 L with a variation of 12.10 (TBW=28±4.37, V=12.10, n=128).

**Table 3:** Variation analysis for the performed 128 measurements in the 32 studied patients by UKM and OCM spKt/V - single pool Kt/V, dialysis adequacy index; URR % - urea reduction ratio, dialysis adequacy index; onKt/V - online Kt/V, dialysis adequacy index, measured by ionic dialysis; TBW - total body water; UKM - urea kinetic modeling; OCM - online clearance monitor

Indicator	Mean value	Standard deviation	Variation
spKt/V	1.36	0.16	0.025
URR%	70.54	5.58	31.15
onKt/V	1.25	0.17	0.028
TBW	28	4.37	12.10

**Figure 1:**
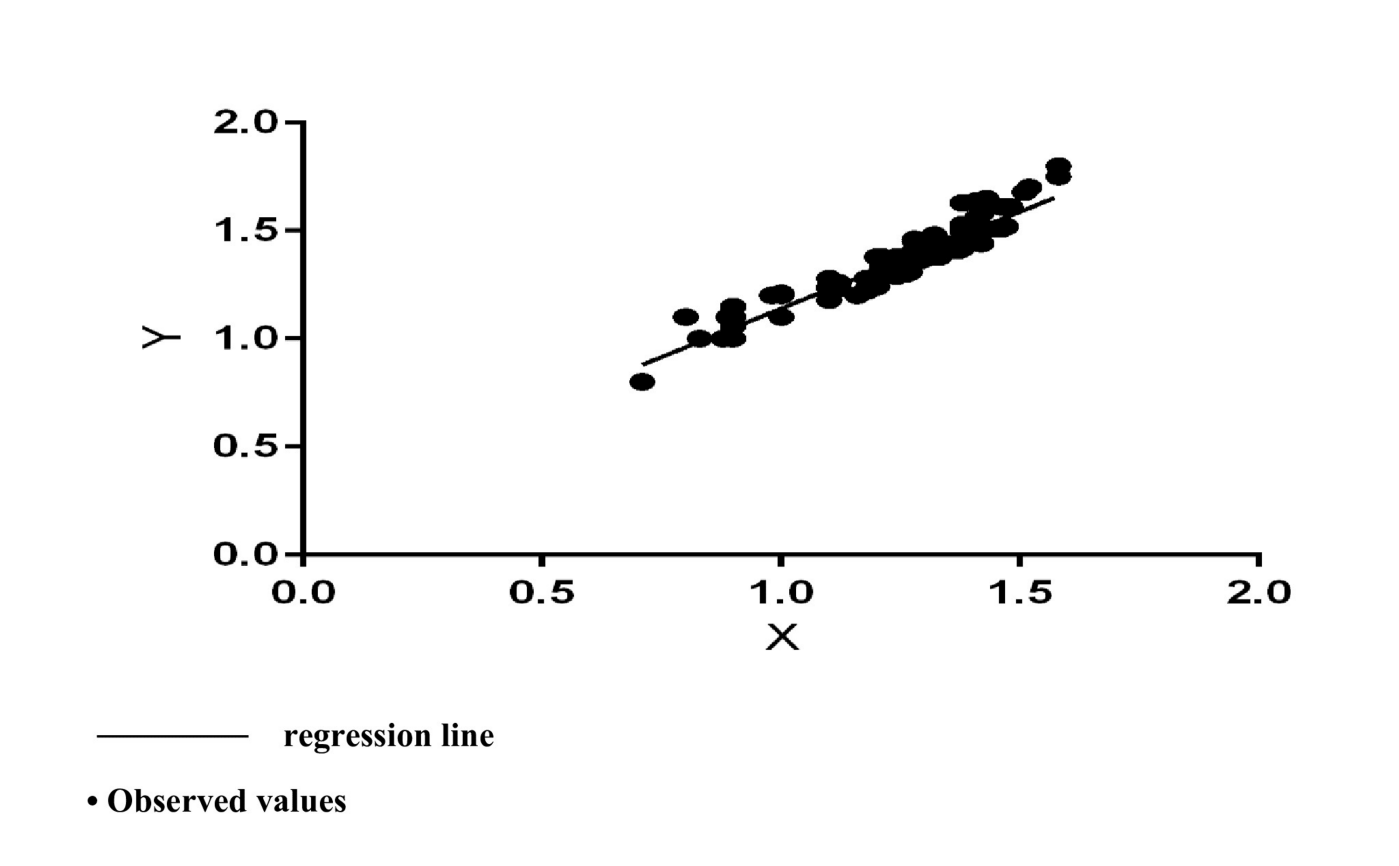
Regression relationship between spKt/V (Y) and onKt/V (X) Linear regression equation: Y=0.8971*X+0.2427, with a statistically significant slope from the horizontal F=991.5, DFn, DFd=1.126, p<0.0001 (R^2^=0.89, Sy.x=0.054)

The obtained results of the Pearson correlation analysis demonstrated a statistically significant correlation between spKt/V and onKt/V values measured by online monitoring (r=0.9419, p<0.00001, R²=0.89). This indicated a strong positive correlation, meaning that high scores for variable X (onKt/V) correspond to high scores for variable Y (spKt/V). Through regression analysis, a straight linear regression was established between the investigated indices (Figure 1). This showed that the measured values of onKt/V had a strong predictive value to spKt/V. The latter can be calculated based on the derived regression equation (Y=0.8971*X+0.2427) without the need to conduct additional blood tests research for verification of the results.

The derived regression model showed that each unit change in onKt/V leads to a change in spKt/V by 0.8971 (95% CI for slope=0.8412-0.9529). The coefficient of determination (R²=0.89) indicated that 89% of the total variance in spKt/V could be explained by the variance of on Kt/V. The remaining 11% was due to factors outside the model, with a standard error of estimate (Sy.x) of 0.054. Despite the high degree of correlation, the fact that the onKt/V values had the ability to underestimate the spKt/V value should not be overlooked. The measured onKt/V values were 8% lower. Student's t-test for dependent variables showed a statistically significant difference between the two indicators (t=-23.031832, p<0.00001).

**Figure 2:**
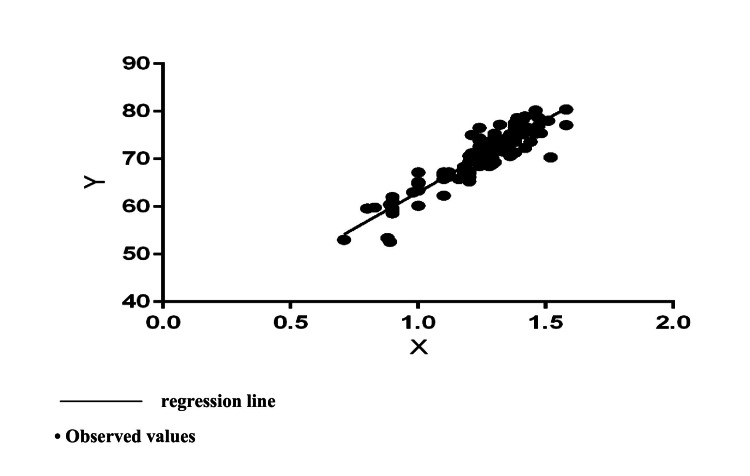
Regression relationship between onKt/v (X) and URR% (Y) Linear regression equation: Y=30.43*X+32.58, with a statistically significant slope from the horizontal F=618.0, DFn, DFd=1.126, p<0.0001 (R^2^=0.83, Sy.x=2.306)

A strong correlation was also established for the measured values of URR% (r=0.9114, p<0.00001, R²=0.83). The regression analysis demonstreated a straight linear regression (Figure 2) between the studied indicators with a regression equation: Y=30.43*X+32.58.

The derived model and its coefficients (F=618.0, R²=0.83, p<0.0001) reflect the statistical significance of the relationship, with a regression coefficient of 30.43 (95% CI for Slope=28.04-32.83). Despite the high coefficient of determination (83%), the calculation of URR% based on ionic dialysis had less clinical significance due to a number of shortcomings of URR% as an indicator for evaluating dialysis adequacy. Nevertheless, the established linear regression between onKt/V recorded by ionic dialysis and the classic indicators of adequacy obtained on the basis of UKM - spKt/V and URR% enabled monitoring of the received dialysis dose in each dialysis session and its adaptation according to the individual needs of the particular patient.

## Discussion

The analysis of the results obtained by us proves a high correlation between the validated indicators of dialysis adequacy (spKt/V; URR) and those registered with online monitoring by ionic dialysis (onKt/V), while at the same time reporting a significant difference between the two methods based mainly on of the anthropometric formulas used to estimate the volume. This finding has been objectified by other authors in previous publications [3,5,6,9,12-20]. Despite the high bond strength, our recorded onKt/V values ​​are 8% lower than the results using UKM. Therefore, onKt/V as an assessment metric has the ability to underestimate the dialysis dose received. Various factors as a possible source of error and its cause have been reported by some authors. Several studies have reported differences of 2-5% in instantaneous conductance measurements, which they associate mainly with differences in the diffusion coefficients of urea and sodium, as well as different effects of dialysis membrane charge or inadequate ultrafiltration correction [1,16]. According to Gotch et al. (2004), spKt/V may be underestimated due to the effects of systemic sodium loading during measurements, resulting in a reduced conductance diffusion gradient across the dialyzer, particularly at urea clearance higher than 150 ml/min [21].

To ensure maximum comparability of the result, our study was conducted while eliminating possible additional factors influencing the received dialysis dose, such as permeability and dialyzer surface area, blood flow rate-Qb and dialysate flow rate-Qd, problematic vascular access with impaired flow and/or recirculation, HD duration, sodium profiling (possible conductance interferences). The measurements were performed within the same session, i.e., each of the above factors would affect both methodologies equally. As the main reason for the underestimation of the received dialysis dose at onKt/V, we assumed the use of anthropometric formulas for estimating the urea volume of distribution (V) - Watson's formula for total body water. It should be kept in mind that the latter is derived on the basis of anthropometric data from healthy individuals and may overestimate total body water in chronodialysis patients due to depletion of muscle mass and impaired nutritional status, as well as not accounting for postdialysis urea rebound - opinion, confirmed by other authors [4,9,15,17,20,22]. Studies by McIntyre et al. (2003) and Alayoud et al. (2012) assumed that the overestimation of V with anthropometric formulas was the reason why ionic dialysance showed better agreement with two-space modeling - double pool Kt/V (dpKt/V), which contradicted the results of Di Filippo et al. (2001) who demonstrated significantly higher values ​​for spKt/V measured by ionic dialysance compared to dpKt/V [4,13,23]. This inconsistency in reported results may be explained by differences in the characteristics of the study populations and the nature of the conductance methods used [6,18]. To estimate the urea volume of distribution (Vukm), urea-kinetic modeling can also be used to indirectly calculate a theoretical urea clearance, which may, however, be significantly lower than the actual one. Reasons for this are most often access recirculation, low blood flow, as well as suboptimal heparinization with thrombosis of capillary fibers in the dialyzer. Therefore, this method requires strict tracking and error correction in one-dimensional modeling [4,16,17]. The high agreement between anthropometrically calculated V and Vukm reported by some authors can be explained by the use of the theoretical clearance - KoA of the dialyzer, which is factory set, and not the effective urea clearance. This leads to an overestimation of its efficiency in vivo and creates conditions for error in the estimation of V, similar to Watson's formula [3,16,21,24]. According to Wuepper et al. (2003), Vukm was significantly higher than the actual volume of distribution of urea as well as that measured by bioimpedance -Vimp, while data from Koubaa et al. (2010) reported that Vukm demonstrated a high correlation with the same [16,22]. Despite the encouraging results, it should not be overlooked that the Vukm score can be affected by errors in blood sampling, the rate of urea rebound, as well as residual renal function [17]. According to Alayoud et al. (2012) adjusting for these factors makes Vukm much closer to Vimp. At the moment, bioimpedance shows the best agreement with TBW, respectively, with the actual urea volume of distribution; however, the results differ depending on the applied impedance frequency and also depending on the position and contact of the electrode [4,20].

Our results demonstrate a very good agreement of onKt/V with the KDOQI-validated indicator of received dialysis dose - spKt/V [2]. Despite the registered statistically significant difference between the two indicators, the strength of correlation between them is very high (r=0.91, p<0.001). Similar data has been reported in recent studies by Rodriguez et al. (2021), Churchill et al. (2021), Raiman et al. (2020), Mohamed et al. (2018), Creput et al. (2013) and Locatelli et al. (2013) [5,11,12,19,20,25]. Our study confirmed the findings of other authors that OCM has the ability to underestimate the dialysis dose received - onKt/V results were 8% lower compared to spKt/V. It is theoretically possible that this difference is due to the convective transport of sodium during the dialysis procedure, which would also lead to a change in the reported conductance and result interference. This is due to the fact that the OCM methodology does not take into account convection, unlike the UKM [18,21,26].

Regardless of any shortcomings of the method, its high degree of correlation with the received dialysis dose is promising for new perspectives on the concept of dialysis adequacy and optimal clinical outcome. Even underestimating to a certain extent the received dialysis dose, online monitoring enables the assessment of dialysis adequacy in real time without the need for blood tests before and after the dialysis session. This demonstrates the advantage of the method compared to the classic UKM - on the one hand, in a purely financial aspect, and on the other, which is undoubtedly of much greater importance, is the strict quality control of the dialysis dose. Monitoring it at longer time intervals in routine practice creates a prerequisite for missing important events, leading to inadequate dialysis and worsening the outcome.

Effective ionic dialysis provides a reliable, real-time, and inexpensive measurement of dialysis dose during an ongoing dialysis session. It allows the clinician to take the necessary interventions, as well as to immediately assess their impact [6]. Thus, for example, with data on a much higher than prescribed dialysis dose received, respectively early reaching the last one in one to two hours in patients without accompanying malnutrition (reduction in V volume) with a normal or even a high body mass index, one should think for access recirculation, even in the absence of clinical data on this. The reason for this phenomenon is that the greater the recirculation of the extracorporeal circuit, the faster the concentration of uremic toxins and, accordingly, that of sodium required for OCM is reduced [6,7]. In other cases, OCM registers persistently low results for dialysis clearance, which are a sign of inadequate prescriptions, problematic access, or inadequate anticoagulation with the risk of clotting in the extracorporeal system - factors that can be recognized early and with any procedure.

Limitations

The study has some limitations such as its retrospective nature, small sample size (due to the small number of devices providing OCM), the use of antrometric formulas to estimate volume instead of bioimpedance, the large interval of blood sampling, which is regulated by budget and the National Health Insurance Fund of the Republic of Bulgaria, the inclusion of patients only aged 18 and over. Оn the other hand, the small number of participants in the study and its short duration were limited by the requirement for an efficiently functioning permanent vascular access. Failure to comply with the latter condition would significantly bias the result, which is why some patients were excluded from the sample.

## Conclusions

This study confirms that OCM is a practical tool for daily use that complements the classic performance of UKM by helping to deliver an adequate dialysis dose with increased patient benefit and minimal cost of financial resources. The reported difference of 8% is negligibly low in clinical terms, especially given the ability to monitor the dialysis dose at each session without the need for blood tests. Our opinion is that with future research, development, and implementation of OCM in routine clinical practice, an opportunity is created for strict control of dialysis adequacy, timely recognition of the causes of inadequate dialysis, and therapeutic intervention on the factors causing the worsening result. However, it must be emphasized that the clinical picture of the patient is above any formula and methodology and should be the leading one in the prescription of dialysis adequacy.
